# Neur-Ally: a deep learning model for regulatory variant prediction based on genomic and epigenomic features in brain and its validation in certain neurological disorders

**DOI:** 10.1093/nargab/lqaf080

**Published:** 2025-06-13

**Authors:** Anil Prakash, Moinak Banerjee

**Affiliations:** Human Molecular Genetics Lab, Neurobiology and Genetics Division, BRIC-Rajiv Gandhi Centre for Biotechnology, Thiruvananthapuram, Kerala 695014, India; Department of Biotechnology, University of Kerala, Kariavattom, Thiruvananthapuram, Kerala 695581, India; Human Molecular Genetics Lab, Neurobiology and Genetics Division, BRIC-Rajiv Gandhi Centre for Biotechnology, Thiruvananthapuram, Kerala 695014, India

## Abstract

Large-scale quantitative studies have identified significant genetic associations for various neurological disorders. Expression quantitative trait locus (eQTL) studies have shown the effect of single-nucleotide polymorphisms (SNPs) on the differential expression of genes in brain tissues. However, a large majority of the associations are contributed by SNPs in the noncoding regions that can have significant regulatory function but are often ignored. Besides, mutations that are in high linkage disequilibrium with actual regulatory SNPs will also show significant associations. Therefore, it is important to differentiate a regulatory noncoding SNP with a nonregulatory one. To resolve this, we developed a deep learning model named Neur-Ally, which was trained on epigenomic datasets from nervous tissue and cell line samples. The model predicts differential occurrence of regulatory features like chromatin accessibility, histone modifications, and transcription factor binding on genomic regions using DNA sequence as input. The model was used to predict the regulatory effect of neurological condition-specific noncoding SNPs using *in silico* mutagenesis. The effect of associated SNPs reported in genome-wide association studies of neurological condition, brain eQTLs, autism spectrum disorder, and reported probable regulatory SNPs in neurological conditions were predicted by Neur-Ally.

## Introduction

Understanding the genetics of complex disorders is extremely difficult due to the heterogeneity within and between populations [1]. The decreasing cost of sequencing and the emergence of custom genotyping arrays have resulted in an increase of quantitative genetic studies like genome-wide association studies (GWAS) and expression quantitative trait locus (eQTL) analysis. Linkage disequilibrium between regulatory and normal single-nucleotide polymorphisms (SNPs) can lead to the emergence of non-causative genetic variants in association results [2]. Identifying the actual causative variant using experimental techniques will be extremely difficult. The common significant variant may be having a narrow effect on the phenotype and the combined effect of multiple variants will be needed for the phenotype to occur [2]. So, functional screening of a large number of associated single genetic variants will be challenging.

Noncoding genetic variants are highly enriched in risk variants, identified by quantitative genetic analysis of complex diseases [3]. The effect of such mutations can be indirect and regulatory in function. The effect of coding mutations like missense and nonsense SNPs can be studied by analyzing the structural changes introduced in the normal protein. In contrast, the regulatory effect of a noncoding SNP will be difficult to decipher. The regulatory landscape can be cell line- or tissue-specific and this adds to the problem. Large number of epigenomic datasets from different tissues and cell lines are available from the Encode project [4]. The incorporation of regulatory features improved the modeling and representation of complex diseases [5]. Hence, the use of tissue specific regulatory datasets will aid in understanding more about the interplay between the regulome and complex diseases.

Through the use of ChIP-Seq, researchers have made substantial progress in locating transcription factor (TF) binding sites, investigating the regulatory roles of TFs in gene expression, and mapping histone modifications throughout the genome [6]. ChIP-Seq analysis has increasingly been combined with other functional genomics techniques, facilitating a deeper understanding of the mechanisms that regulate gene expression [7]. A wide array of quantitative methodologies has significantly contributed to the progress in assessing chromatin accessibility. Continuous improvements in integrated analysis are expected to enhance our comprehension of the intricate relationships among DNA accessibility, gene expression, genetic variants, protein interactions, transcription, and subsequent phenotypes [8].

The regulation of genes, which includes transcription and alternative splicing, is fundamentally influenced by DNA- and RNA-binding proteins. Recent advancements in deep learning methodologies have facilitated the prediction of the sequence specificities of these proteins, thereby enhancing our understanding of regulatory mechanisms [9]. Deep learning models are capable of discerning regulatory sequence patterns from extensive regulatory datasets, such as chromatin accessibility data. This capability allows for the prediction of chromatin effects resulting from sequence modifications with single-nucleotide precision. As a result, these models have significantly improved the prioritization of functional variants, which encompasses eQTLs and variants linked to diseases [10].

Computational tools that are created using cell- or tissue-specific datasets will help in better understanding of the diseases that are connected to those types of tissues or cells. This prompted us to create a model for all neurological conditions, which can be trained on neuronal -specific epigenomic datasets and that will subsequently help in the variant effect prediction of mutations specific to neurological conditions. Several deep learning models have been developed with applications in biology [11]. They will learn the linear and nonlinear relationships within the vast amount of data that are available. Attention layers have improved the performance of various natural language processing (NLP) tasks that in turn helped in the analysis of sequence datasets [12]. With this in mind, we developed a deep learning model called Neur-Ally. The model has convolution and attention layers incorporated into the architecture.

The model after training can be used to predict the regulatory effect of SNPs specific to neurological conditions. These SNPs can be chosen from significant GWAS associations or candidate genetic association studies for neurological conditions [13]. Alternatively, eQTL SNPs that regulate the expression of genes can also be used for the prediction [14]. In case of variants occurring in non-neuronal tissues contributing to the phenotype, epigenomic datasets from those samples can also be processed using the data processing codes available with the model. For neurological condition-specific mutations, the pretrained weights along with the codes are available for testing. In short, Neur-Ally will help in identifying the regulatory potential of SNPs in the brain, based on the differential epigenomic signatures in response to *in silico* mutagenesis.

## Materials and methods

### Model architecture

The genomic bins where epigenomic labels overlap are used as input to the model. The 200-bp sequence of the bin along with the flanking regions (1800 bp) is subjected to vectorization, word embedding, and positional encoding to create a multidimensional tensor. In contrast to words in natural language, which have semantic associations, genomic sequences are categorical (A, C, G, T, N). By treating each nucleotide as a separate category, the integer mapping avoids making assumptions about biological similarities. Since reference genomes frequently contain ambiguous regions, it is imperative that N (unknown base) be included in genomic data. The model can differentiate between these positions during processing (e.g. through masking) by mapping N to 0. Because genomic sequences might be lengthy (in this case, 2000 bp) and just a tiny integer is needed at each point, using np.int8 maximizes memory use.

Nucleotide embeddings are taught to capture task-relevant context-specific patterns, such as binding site prediction, in contrast to NLP, where embeddings capture semantic associations. Positional encoding adds each nucleotide’s position in the sequence to its embedding. Because genomic sequences are ordered and a nucleotide’s position (e.g. relative to a regulatory element) may influence its functional role, this is significant. Positional encoding uses sine and cosine functions to generate a positional encoding matrix in accordance with the transformer model’s methodology [15]. The mask scales the positional encodings to ignore N positions before appending them to the input embeddings.

For each position *p* ∈ {0, 1, …, seqlen − 1} and dimension *d* ∈ {0, 1, …, dim − 1}, the encoding is PE(*p*, 2*i*) = sin(*p*/10 000^2*i*/dim^) and PE(*p*, 2*i* + 1) = cos(*p*/10 000^2*i*/dim^).

The choice of the embedding dimension (dim = 128) is a compromise between computational efficiency and expressiveness. Then, it is fed into subsequent layers of 1D convolution and max pooling twice, followed by multi-head attention (MHA) layers. A kernel size of 10 nucleotides is chosen to capture short genomic patterns, such as TF binding sites, which typically span 6–20 bp.

The first layer uses 64 filters to learn a large number of motifs, while the second layer uses 32 filters to save processing costs and concentrate on higher-level combinations of these motifs. By capturing all possible windows in the sequence, the convolution with a stride of 1 maximizes the recognition of motifs regardless of their exact placement. Pooling reduces the sequence length by a factor of 3, focusing on the strongest motif signals and reduces computational complexity. The stride of 3 ensures nonoverlapping pooling, further reducing the complexity.

For a 1D convolution with input *X* ∈ *R*batch size × seqlen × dim, weights *W* ∈ *R*kernel size × dim × filters, and bias *b* ∈ *R*^filters^:


\begin{eqnarray*}
&&{{Y}}\left[ {{i, t, f}} \right] \nonumber \\ &&=\, {\mathrm{ReLU}}\left(\sum\nolimits_{{{(k = 0)}}}^{{\mathrm{(kernel\, size - 1)}}} {\sum\nolimits_{{{d = 0}}}^{{\mathrm{dim - 1}}} {{{W}}\left[ {{k, d, f}} \right]} \,{{X}}\left[ {{i, t + k, d}} \right]{{ + b}}\left[ {f} \right]}\right){\rm },
\end{eqnarray*}

where *t* is the output position, *f* is the filter index, and output *Y* ∈ *R*batch size × (seqlen − kernel size + 1) × filters.

For max pooling with pool size *p* and stride *s*, *Z*[*i*, *t*, *f*] = ${\mathrm{max}}_{{{k}} = 0}^{p - 1}$*Y*[*i*, *t* ⋅ *s* + *k*, *f*], where output *Z* ∈ *R*batch size × ⌈(seqlen − kernel size + 1)/*s*⌉ × filters.

Long-range dependencies between nucleotides can be captured by the model by using MHA, which enables it to focus on multiple sequence segments at once. This is helpful for genomic data, where regulatory elements may be separated by hundreds of base pairs.

- Compute query (*Q*), key (*K*), and value (*V*) matrices: *Q* = *XW*_*Q*_+ *b*_*Q*_, *K* = *XW*_*K*_+ *b*_*K*_, and *V* = *XW*_*V*_+ *b*_V_.
- Split *Q*, *K*, and *V* into heads along the dimension axis.
- Compute scaled dot-product attention for each head: attention(*Q*, *K*, *V*) = softmax(*QK*^T^/√*d*_*k*_)*V*, where *d_k_*= dim′/heads is the dimension per head.

The squeeze operation, *Y* = squeeze(*Y*, axis = −1), is a lightweight alternative to global pooling to reduce the tensor to a manageable size for the final dense layer.

**Figure 1.**
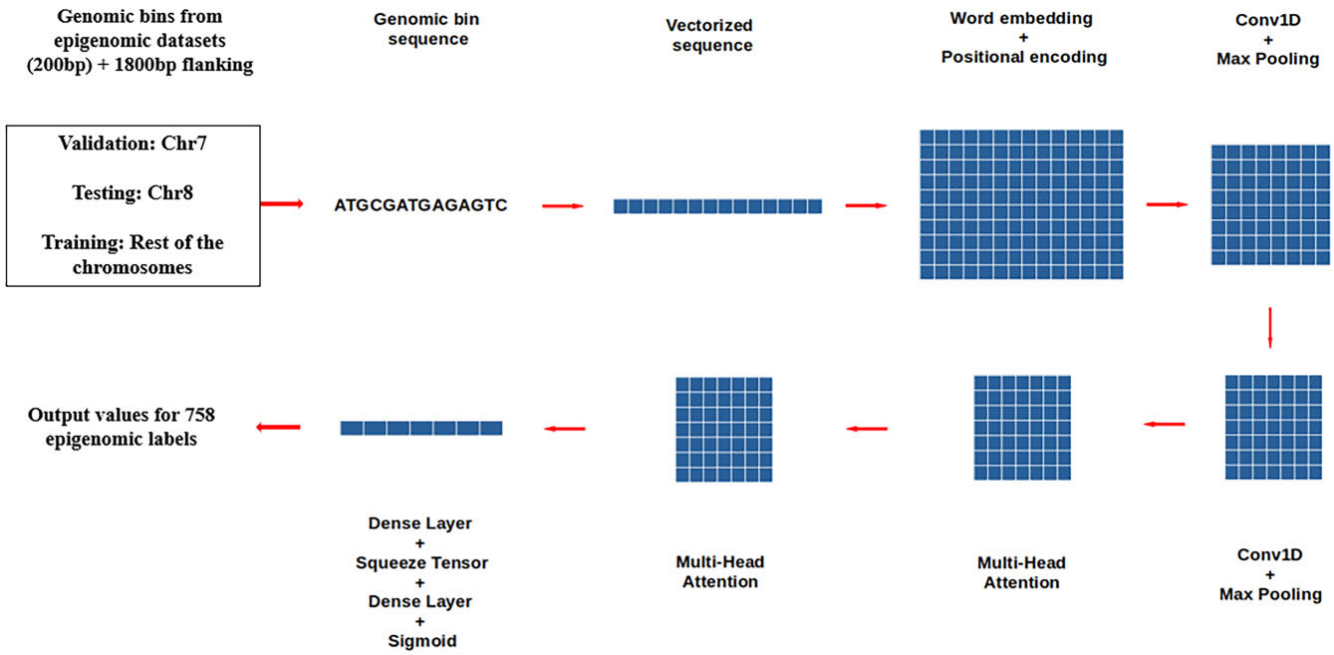
Neur-Ally architecture. Flowchart diagram of the model architecture.

The output from the final attention layer is fed into the Keras dense layer [16]; this is followed by reduction in the dimension of the tensor using squeeze operation. The final dense layer and sigmoid activation function provide the output of the probability of regulatory signatures in the genomic bin region used as the input. The flow diagram of the model architecture is shown in Fig. 1.

### Data processing

**Figure 2.**
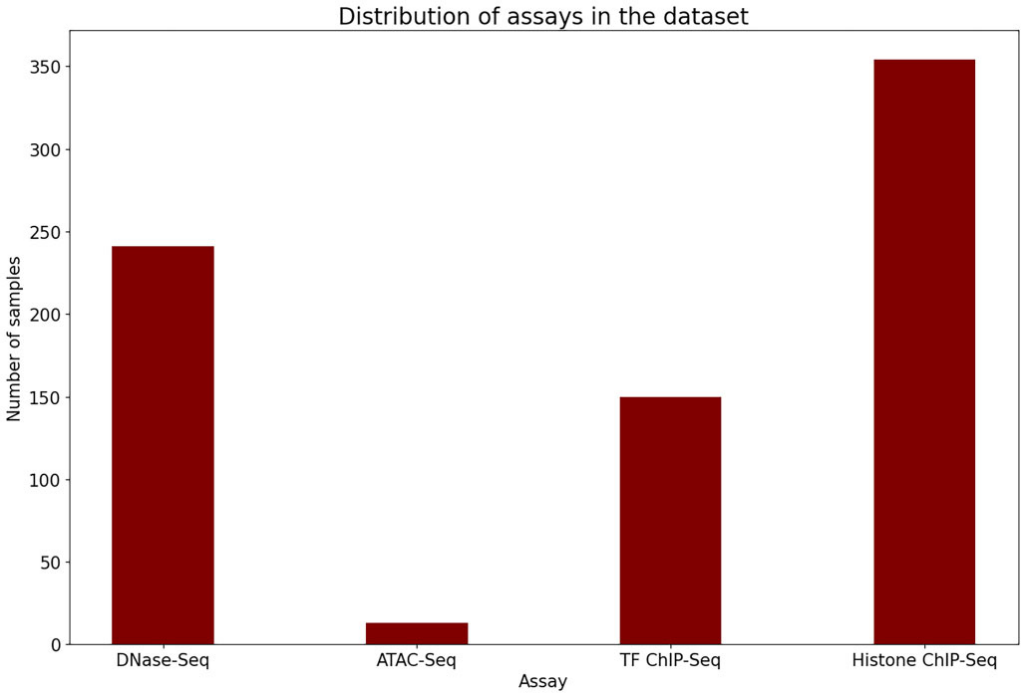
Assay distribution. Bar graph of assays used for data preprocessing.

Epigenomic datasets regarding chromatin accessibility (ATAC-Seq, DNase-Seq), histone modifications, and TF binding pertaining to tissue type or cell type were selected for processing the data (Fig. 2). The narrow peak bed files of nervous tissue and cell samples were extracted from the Encode project. Genomic bins of 200 bp length were selected as positive samples if the epigenomic signature is overlapping more than half of it. As the model output is multilabel, it predicts several labels or categories for a single input. Each label is independent and has the potential to be true or false at the same time. Therefore, the terms “positive” and “negative” samples describe whether or not particular labels are present in a particular instance. Genomic bins with low mappability were excluded from the analysis. The processed dataset was split into training, validation, and testing based on the chromosome number of the genomic bin. Those belonging to chromosome 7 and chromosome 8 were used for validation and testing, whereas the remaining ones were kept for training. For testing the model predictions, area under the curve of receiver operator characteristic (AUROC) and precision recall area under the curve (PR-AUC) were estimated. The baseline PR-AUC is based on positive class prevalence, meaning it reflects the performance of a model that predicts the positive class with a probability equal to the proportion of positive samples in the dataset.

For a dataset with *N*_+_ positive samples and *N*_−_ negative samples, the positive class prevalence is: *p* = *N*_+_/(*N*_+_ + *N*_−_).

### Variant effect prediction

As the model was trained on genomic sequence and regulatory labels, it can learn the contribution of sequence features to the prediction. Thus, the prediction of regulatory labels upon giving altered sequences as input can shed light on the regulatory effect of mutations. Therefore, we predicted the regulatory effect of SNPs specific to neurological conditions by *in silico* mutagenesis. The sequence of the genomic bin harboring the mutation was extracted from the reference genome. Another input sequence was generated by altering the nucleotide at the mutation site. Both the inputs were given to the model in a sequential manner. The predictions of the regulatory labels were compared for both the sequences to estimate the SNP activity difference (SAD) score [17].


\begin{eqnarray*}
{\mathrm{SAD = |}}{{{{P}}}_{{\mathrm{ref}}}}{\mathrm{ - }}{{{{P}}}_{{\mathrm{alt}}}}{\mathrm{|,}}
\end{eqnarray*}

where *P*_ref_ is the probability of regulatory labels predicted on the reference sequence, whereas *P*_alt_ is the probability of regulatory labels predicted on the mutated sequence. SAD score is the absolute difference between *P*_ref_ and *P*_alt_.

To identify significant regulatory variants, we created a negative nonregulatory set of SNPs from the 1000 Genomes dataset [18]. A million variants were randomly selected and a negative set of SNPs was created by filtering GWAS and eQTL variants and those occurring in exonic or candidate *cis-*regulatory regions. The significance of the regulatory effect of the predicted variants was estimated using the *E*-value method [19]. *E*-value of an epigenomic target for a particular SNP is defined as the ratio of SNPs from the negative nonregulatory set having higher SAD score for the same target. The same number of positive and negative variant sets are used for *E*-value prediction. The selection of negative samples is repeated 10 times and the mean *E*-value is selected for comparison. SNPs with an *E*-value of 1e−05 or less are considered as significant.

Neur-Ally was trained on epigenomic datasets with coordinates according to the hg38 human reference build. So, the input variant coordinates have to be based on the hg38 build. While using SNP datasets belonging to older genomic builds, the coordinates were converted to the latest genomic build using liftover tools. Chromosomal coordinates belonging to conversion unstable positions were excluded from the analysis [20].

### Model prediction on GWAS of neurological conditions and eQTL SNPs

The trained model was used to identify the differential regulatory label prediction of neurological condition-specific SNPs extracted from the GWAS catalog and eQTL variants in the brain tissues. For the neurological condition-specific GWAS SNPs, “GWAS catalog v1.0” dataset was extracted and associated SNPs were selected based on matching keywords in the disease or trait column. The following keywords were used for the filtering: “alzheimer,” “epilepsy,” “multiple sclerosis,” “parkinson,” “autism,” “attention deficit,” “schizophrenia,” “bipolar,” and “major depressive.” Significant variant–gene pair datasets of the neuronal tissues from the GTEx portal were used for selecting the neurological condition-specific eQTLs. Top 1000 significant eQTL SNPs from each sample were used for creating the list of eQTLs to be tested.

### Model prediction on ASD GWAS and brain regulatory SNPs

The *E*-value threshold of 1e−05 is a stringent one, but since the calculated *E*-value will depend on the number of variants present in the positive set, so, we tried to restrict the prediction to autism spectrum disorder (ASD)-associated SNPs from the GWAS catalog. Hence, we used the keywords, “Asperger disorder,” “Autism,” and “Autism spectrum disorder,” to select the variants from the dataset. Next, we wanted to test the model performance on reported probable brain regulatory SNPs [21]. As the number of positive variants is few, 200 negative variants were sub-sampled for comparing the SAD scores.

## Results

### Model performance

**Figure 3.**
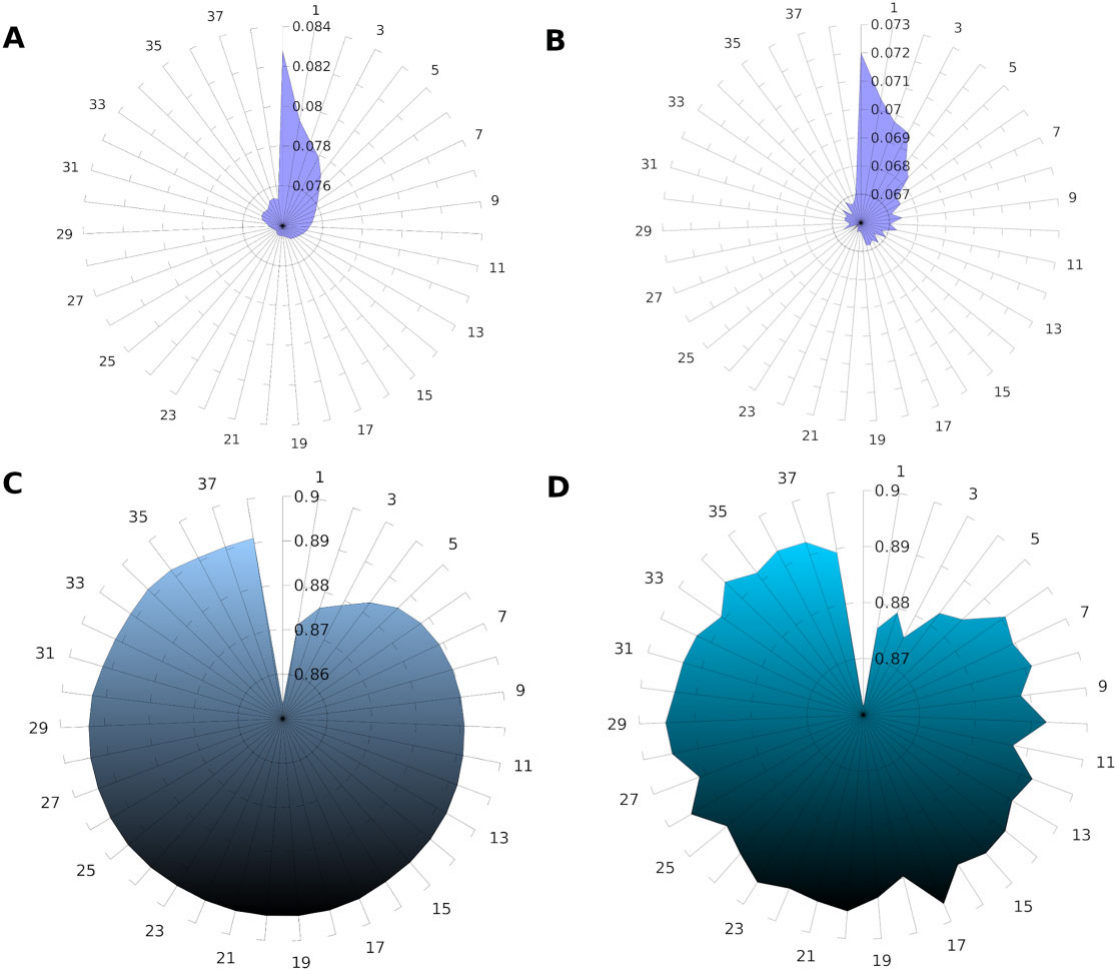
Training and validation metrics. Radar plots showing (**A**) training loss, (**B**) validation loss, (**C**) training mean AUROC, and (**D**) validation mean AUROC over subsequent epochs. Epochs are depicted in circular axes and metric or loss values in radial axes.

The binary cross-entropy loss values and metrics during training and validation over 39 epochs are depicted in Fig. 3. The metric values generated by Keras were approximated ones and the individual metric values of each epigenomic label were generated using scikit-learn [22] (Supplementary Table S1). The prediction of chromatin accessibility assay labels had a mean AUROC of 0.93 and PR-AUC of 0.23 (baseline PR-AUC is 0.01). Histone modifications had a mean AUROC of 0.84 and PR-AUC of 0.29 (baseline PR-AUC is 0.03). TF binding labels had a mean AUROC of 0.87 and PR-AUC of 0.22 (baseline PR-AUC is 0.01).

### GWAS and neurological condition-specific eQTL variants

**Figure 4.**
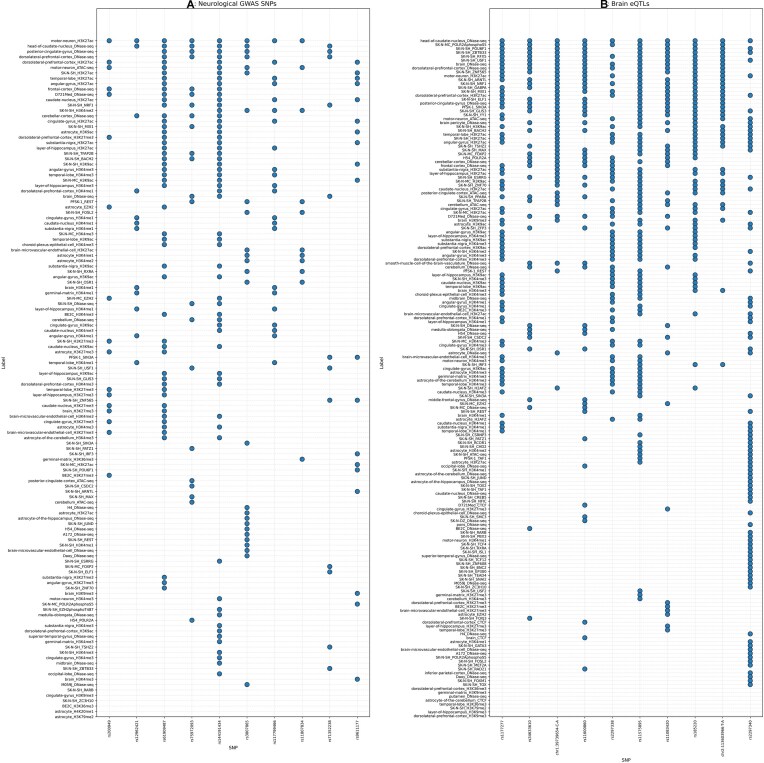
Dot plot. Top 10 variants with a greater number of significant regulatory labels. (**A**) Neurological condition-specific GWAS SNPs. (**B**) Neurological eQTL SNPs. Variants in *x*-axis and labels in *y*-axis.

GWAS-associated SNPs were selected after removing GWAS catalog variants occurring in the coding regions. Seven thousand six hundred and sixty-three neurological condition-specific SNPs were extracted using keywords and selected as the positive variant set. Forty-eight SNPs were showing significant *E*-values after comparing with the negative variant set at a threshold of 1e−05 (Supplementary Table S2). The top variants with a greater number of significant regulatory labels are shown in Fig. 4A. Using a less stringent threshold can reveal more possible regulatory variants.

The significant gene–variant pair files (v8) of the following samples belonging to the nervous system from GTEx portal were used for extracting neurological condition-specific eQTL positive variant set: “Cerebellum,” “Nucleus accumbens basal ganglia,” “Cortex,” “Caudate basal ganglia,” “Cerebellar Hemisphere,” “Anterior cingulate cortex BA24,” “Amygdala,” “Spinal cord cervical C-1,” “Hypothalamus,” “Substantia nigra,” “Frontal Cortex BA9,” “Hippocampus,” and “Putamen basal ganglia.” Top 1000 hits from each sample were used for the predictions, and after stringent filtering, 169 highly significant regulatory SNPs were predicted by the model (Supplementary Table S3). The top variants with a greater number of significant regulatory labels are shown in Fig. 4B.

### ASD GWAS variants

**Figure 5.**
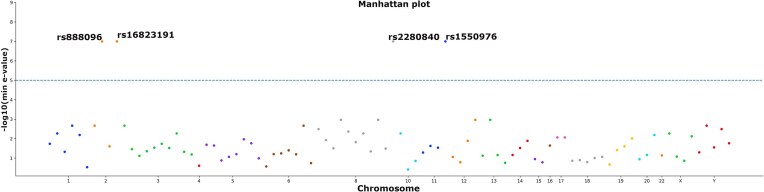
Manhattan plot of ASD regulatory variants predicted by Neur-Ally. ASD associated SNPs from GWAS catalog having significant *E*-values are shown above the horizontal threshold line.

The variant effect predictions were restricted to 92 ASD SNPs from GWAS catalog and 4 highly significant regulatory ones were predicted by the model (Fig. 5). The significant labels and their *E*-values are shown in Supplementary Table S4.

### Regulatory brain variants

The model was tested for variant effect prediction on reported probable regulatory variants in the brain (Table 1). Eight such SNPs were selected from publications [23–29] and their differential epigenomic changes upon *in silico* mutagenesis were predicted by the model (Table 1). The annotation of the variants from VARAdb [30] is reported in Table 2. Predictions of differential epigenomic labels for rs7364180 and rs12411216 were found to be significant. rs7364180 is found to be associated with many eQTL genes in brain tissues [28]. rs12411216 is reported to be a probable regulatory risk variant for mild cognitive impairment in Parkinson’s disease [29]. rs7364180 is suggested to be involved in the formation of chromatin loops via CCCTC-binding factor (CTCF) that could regulate the expression levels of eQTL genes in cooperation with nearby enhancer regions. For the SNP rs7364180, the model predicted significant chromatin accessibility changes in samples like motor neuron, head of caudate nucleus, brain microvascular endothelial cell, and cell lines like SK-N-SH, H54, Daoy, and A172. In addition to that, significant changes were also found in TF ChiP-Seq labels from dorsolateral prefrontal cortex, PFSK-1, and SK-N-SH. Neur-Ally also predicted significant changes in chromatin accessibility because of rs12411216 in dorsolateral prefrontal cortex and posterior cingulate gyrus.

**Table 1.** Neur-Ally predictions for reported probable brain regulatory variants

SNP^a^	Functional effect^b^	Min. *E*-value^c^	Min. *E*-value Label^d^
rs405509	Decreased APOE expression in brain tissue [17]	0.0115	Layer of hippocampus (H3K27me3)
rs2526377	Increased expression of PICALM and TGFBR1 in neuronal progenitor cells [18]	0.016	Astrocyte (H4K20me1)
rs6733839	BIN1 depletion in microglia [19]	0.0649	Motor neuron (H3K27ac)
rs1990620	Frontotemporal dementia risk, increases CTCF recruitment [20]	0.0005	Dorsolateral prefrontal cortex (chromatin accessibility)
rs356168	Parkinson’s disease risk, regulates SNCA gene expression [21]	0.004	SK-N-SH (ZC3H10 ChiP-Seq)
rs1476679	Alzheimer’s disease risk, associated with eQTL genes [22]	0.0005	SK-N-SH (POLR2AphosphoS5 ChiP-Seq)
rs7364180	Alzheimer’s disease risk, associated with eQTL genes [22]	0.0	Dorsolateral prefrontal cortex (CTCF binding)
rs12411216	Parkinson’s disease risk, regulates glucocerebrosidase expression [23]	0.0	Dorsolateral prefrontal cortex (chromatin accessibility)

^a^rsIDs of the regulatory SNPs.

^b^Functional effect reported in the publications and their reference number.

^c^Minimum *E*-value predicted by the model.

^d^Epigenomic label having the lowest *E*-value.

**Table 2.** Annotation information from VARAdb: a comprehensive variation annotation database for human

rsID	N_enhancer	N_promoter	N_ATAC	N_eQTL	Major associated phenotype
rs12411216	337	116	56	18	Occipital lobe volume
rs6733839	40	0	7	15	Alzheimer’s disease
rs2526377	157	51	27	3	Platelet hematocrit measurement
rs405509	58	37	15	2	Alzheimer’s disease
rs1476679	19	0	2	9	Alzheimer’s disease
rs7364180	76	0	11	9	Alzheimer’s disease
rs1990620	3	0	7	8	–
rs356168	234	0	22	1	Parkinson’s disease

rsID: reference SNP identifier from dbSNP 151; N_enhancer: count of enhancers and super-enhancers containing the variant; N_promoter: count of promoters containing the variant; N_ATAC: count of accessible chromatin regions from TCGA and cistrome containing the variant; N_eQTL: count of eQTL pairs associated with the variant.

## Discussion

Quantitative genetic studies like GWAS and eQTL analysis in neurological disorders have revealed several risk variants in the noncoding regions of the genome. The functional consequences of the coding variants can be interpreted by the effect of the mutation on the protein structure. But for noncoding variants, the regulatory consequence can be specific to the cell or tissue type. Experimental methods to determine the effect of all the significant variants from a study will be difficult because of this heterogeneous nature. Computational tools developed from the publicly available epigenomic datasets can be used to create prediction models for this purpose. Such functional predictions will help to differentiate between actual causative variants and those that are highly linked to them.

In this study, we have created a deep learning model named Neur-Ally; trained it on epigenomic datasets derived from nervous tissues and cells. Most of the existing variant effect prediction models were trained on regulatory datasets from multiple tissues or cell line samples. It will be difficult for such models to learn the regulatory signatures that are cell or tissue specific. In case of diseases where changes in a particular organ or tissue contribute majorly to the pathophysiology, models trained on that particular tissue or cells will be more helpful. The machine learning model trained on human retinal epigenomic datasets was developed to predict the effect of noncoding variants in human retinal *cis-*regulatory elements [31]. Another pancreatic islets specific model trained on multiple epigenome profiling datasets was used for prioritizing type 2 diabetes association signals [32]. The machine learning model for variant effect prediction in alzheimer’s disease was developed and achieved better accuracy compared to other models [33]. The model was trained on 39 features of which 9 were regulatory ones. Data from seven brain tissues were used to form regulatory regions. In contrast, Neur-Ally was developed and trained on multiple brain samples, including tissues, cells, *in vitro* differentiated cells, primary cells, and organoids. Seven hundred and fifty-eight regulatory datasets were used to create a training dataset of 9 Million genomic bins (200 bp) overlapping epigenetic features. Hence, Neur-Ally is important in the sense that it is a variant prediction model that uses a large number of neuronal-specific regulatory datasets and can be used for all neuronal disorders. The model predicts the regulatory labels upon giving nucleotide sequences of genomic bins as input. The model achieved commendable performance while predicting TF binding, histone modifications, and chromatin accessibility upon testing. *In silico* mutagenesis was carried out after training the model and the significant regulatory effects of neurological condition-specific mutations were identified. Regulatory consequences were identified in neurological condition-specific GWAS, eQTL, and ASD GWAS, and reported probable regulatory neurological condition-specific variants.

Immune system abnormalities are identified in patient categories with neurological disorders. Thus, the associated genetic variants may have regulatory functions in non-neuronal samples as well. The data preprocessing scripts available with Neur-Ally can be used for creating training datasets with analysis files of different sample types. For the neuronal predictions, additional epigenomic data can be added whenever they are available. Therefore, the prediction performance of the model that we have developed can further be improvised as and when the newer datasets arrive.

## Supplementary Material

lqaf080_Supplemental_Files

## Data Availability

Neur-Ally is open source and available at https://github.com/anilprakash94/neur_ally and https://doi.org/10.5281/zenodo.14875845. The epigenomic datasets are available at https://www.encodeproject.org/.
