# Supplementary material for: Neur-Ally: a deep learning model for regulatory variant prediction based on genomic and epigenomic features in brain and its validation in certain neurological disorders
Source: NAR Genom Bioinform. 2025 Jun 13;7(2):lqaf080. doi: 10.1093/nargab/lqaf080 (PMC12164584; doi:10.1093/nargab/lqaf080)
Supplement: lqaf080_Supplemental_Files [file lqaf080_supplemental_files.zip › SUPPLEMENTARY INFORMATION.docx]

**SUPPLEMENTARY INFORMATION**

**Supplementary Table S1:** Metric values for individual epigenomic labels. The AUROC and PR_AUC values are given in separate columns and their mean values are provided at the end of the file.

**Supplementary Table S2:** GWAS SNPs of neurological disorders with significant regulatory predictions. The "Signif. labels" column contains the epigenomic labels in which significant regulatory changes were predicted and the respective E-values are given in the "Signif. scores" column.

**Supplementary Table S3:** Top brain eQTL SNPs with significant regulatory predictions. The "Signif. labels" column contains the epigenomic labels in which significant regulatory changes were predicted and the respective E-values are given in the "Signif. scores" column.

**Supplementary Table S4:** Autism Spectrum Disorder GWAS SNPs with significant regulatory predictions. The "Signif. labels" column contains the epigenomic labels in which significant regulatory changes were predicted and the respective E-values are given in the "Signif. scores" column.
